# Associations of Weekday and Weekend Sleep with Children’s Reported Eating in the Absence of Hunger

**DOI:** 10.3390/nu11071658

**Published:** 2019-07-20

**Authors:** Sarah LeMay-Russell, Marian Tanofsky-Kraff, Natasha A. Schvey, Nichole R. Kelly, Lisa M. Shank, Sarah J. Mi, Manuela Jaramillo, Sophie Ramirez, Deborah R. Altman, Sarah G. Rubin, Meghan E. Byrne, Natasha L. Burke, Elisabeth K. Davis, Miranda M. Broadney, Sheila M. Brady, Susan Z. Yanovski, Jack A. Yanovski

**Affiliations:** 1 Department of Medical and Clinical Psychology, Uniformed Services University of the Health Sciences (USUHS), 4301 Jones Bridge Road, Bethesda, MD 20814, USA; 2 Section on Growth and Obesity, Division of Intramural Research, *Eunice Kennedy Shriver National* Institute of Child Health and Human Development (NICHD), National Institutes of Health (NIH), DHHS, 10 Center Drive, Bethesda, MD 20892-1103, USA; 3 Counseling Psychology and Human Services and the Prevention Science Institute, University of Oregon, 1215 University of Oregon, Eugene, OR 97403, USA; 4 Department of Psychology, Fordham University, 411 East Fordham Road, Bronx, NY 10458, USA; 5 Division of Digestive Diseases & Nutrition, National Institute of Diabetes, Digestive and Kidney Diseases, 6707 Democracy Blvd, Rm 6025, Bethesda, MD 20892, USA

**Keywords:** eating in the absence of hunger, sleep, child and adolescent, fat mass, adiposity

## Abstract

Insufficient average sleep duration has been inconsistently associated with poor diet and obesity risks in youth. Inconsistencies in findings across studies may be due to a general failure to examine associations in weekday versus weekend sleep. We hypothesized that greater variations in weekday and weekend sleep duration would be associated with more disinhibited eating behaviors, which, in turn, might be involved in the relationship between sleep and weight. We, therefore, examined, among healthy, non-treatment seeking youth, the associations of average weekly, weekend, and weekday sleep duration with eating in the absence of hunger (EAH), a disinhibited eating behavior associated with disordered eating and obesity. Sleep was assessed via actigraphy for 14 days. Participants completed a self-report measure of EAH. Adiposity was measured by dual-energy X-ray absorptiometry. Linear regressions were used to test the associations of sleep duration with EAH and the associations of sleep duration and EAH, with fat mass. Among 123 participants (8–17 years, 52.0% female, and 30.9% with overweight), there was no significant association between average weekly sleep and EAH. Further, there was no significant association among average weekly sleep duration or EAH and fat mass. However, average weekday sleep was negatively associated, and average weekend sleep was positively associated, with EAH (*p*s < 0.02). Weekend “catch-up” sleep (the difference between weekend and weekday sleep) was positively associated with EAH (*p* < 0.01). Findings indicate that shorter weekday sleep and greater weekend “catch-up” sleep are associated with EAH, which may place youth at risk for the development of excess weight gain over time.

## 1. Introduction

In the United States, over one third of children and adolescents currently have overweight and nearly 20% have obesity [1,2]. Given the negative long-term health impacts of overweight and obesity [3,4,5], the elucidation of early, modifiable risk factors for excess weight gain is necessary to enhance the effectiveness of obesity prevention and treatment in children and adolescents. Two such factors that have been associated with excess weight and adiposity among youth are insufficient sleep [6,7] and disinhibited eating behaviors (eating behaviors characterized by a lack of healthy restraint [8,9,10,11,12,13]).

Sleep plays a critical role in healthy child and adolescent development [14]. Throughout childhood, sleep is developmentally patterned such that as children grow and mature, their sleep needs vary in a predictable manner [15]. Specifically, most research indicates that younger children require more sleep, while older children and adolescents need less [15]. As such, the American Academy of Sleep Medicine recommends the following guidelines for every 24 h of sleep by age: Infants (4 months to 12 months) should sleep 12–16 h, including naps; children ages 1–2 years should sleep 11–14 h, including naps; children ages 3–5 years should sleep 10–13 h, including naps; children 6–12 years should sleep 9–12 h; and adolescents age 13–18 should sleep 8–10 h [16]. Recent data indicate that only 5% of youth get the recommended amount of sleep [17] and that insufficient sleep has been an increasing trend [18]. Children’s sleep duration across all ages is shorter than it was in the past [18], such that the overall duration of sleep among youth in the last 100 years has decreased by approximately one hour per night [18].

Reduced sleep duration has frequently been found to be associated with obesity in childhood and adolescence. Studies generally support a relationship between fewer than eight reported hours of sleep nightly and a greater risk for developing a higher body mass index (BMI), overweight, and obesity [7,19,20,21,22,23]. However, the data are not entirely consistent [22,24]. For example, Jarrin et al. [22] reported that when relevant covariates (such as age, sex, pubertal status, physical activity, screen time, and parental education) were included in their model, the negative association between fewer hours of self-reported sleep and obesity was attenuated and no longer significant. Likewise, Lytle et al. [24] reported no statistically significant prospective relationship between decreases in self-reported sleep and increases in BMI or percent body fat in either adolescent boys or girls.

These mixed results suggest there may be other factors contributing to the association between children’s sleep duration and BMI. One possible consideration is that there are many dimensions of sleep beyond the average number of hours spent in bed [22]. For example, specific characteristics of sleep, such as bedtime shift (i.e., going to bed at different times on weekdays versus weekends) and weekend (Friday and Saturday nights) versus weekday (Sunday through Thursday nights) sleep duration have been associated with a greater severity of overweight and an increased intake of sugar-sweetened beverages [25,26,27]. Again, data are somewhat mixed. For example, Wing et al. found self-reported weekend and holiday “catch up” sleep to be associated with a lower risk of overweight and obesity in children [28].

Given these mixed results, other studies have attempted to elucidate the associations of weekend versus weekday sleep and the neurobiological and behavioral underpinnings that may influence eating and weight [25,29]. For example, Hasler et al. [29] reported that a greater weekend mid-sleep shift and a shift of bedtimes to either earlier or later on the weekend than on weekdays, leading to circadian rhythm misalignment, were associated with a decreased medial prefrontal cortex and a striatal reactivity to reward, suggesting decreased regulatory responses and increased reward sensitivity. It is possible that such a decreased ability to self-regulate could lead to disinhibited eating behaviors, as other studies have shown poor inhibitory control associated with eating in response to external food cues and in response to negative emotional states [30]. Supporting this notion, Ievers-Landis et al. [25] found that later weekend bedtimes compared to weekday bedtimes were associated with decreased eating-related self-efficacy, such that participants had a greater self-reported difficulty regulating negative affect, in order to make healthy food choices (e.g., consuming more whole grains and fruits and vegetables than saturated fats and sugary beverages). These results underscore the need to investigate not only average sleep duration, but also specific sleep characteristics, such as weekday versus weekend sleep and weekend “catch-up” sleep, with subsequent reward-based disinhibited eating behaviors known to be associated with obesity [31].

One disinhibited eating pattern that may play a role in obesity risk is eating in the absence of hunger (EAH), which is characterized by eating when not hungry, or past satiation in response to fatigue, negative affect, or when cued by external circumstances, such as the sight or smell of food [32,33]. Among children, EAH has been shown to be a stable trait [34,35]. EAH also appears to increase with age [34,36], and cross-sectional data have shown that behavior (both measured in the laboratory and by self-report) is positively correlated with fat mass and BMI*z* [32]. However, prospectively, EAH has not been shown to predict increases in BMI, BMI*z*, or fat mass over a 1-year period among youth [32,34,37]. Nevertheless, overweight and obesity track with EAH such that children with overweight and obesity have higher levels of EAH than their counterparts without overweight or obesity, and this relationship persists over time [31]. Although EAH has been shown to be stable, its etiology remains unclear, and it is possible that EAH may initially vary in response to poorer cognitive control and increased reward sensitivity. Given the mixed findings, eating in the absence of hunger (EAH) may put certain individuals at increased risk of excess weight.

Taken together, extant studies suggest that both sleep behavior and EAH have associations with excess weight. However, some studies report inconsistent results, and the relationships among these variables are not fully understood. Further data indicate that facets of sleep, such as weekday versus weekend sleep and weekend “catch-up” sleep, are associated with decreased eating-related cognitive control and increased reward sensitivity [25,30,38,39] that could lead to poorer dietary decisions, including disinhibited eating patterns, such as EAH. There are some data among adult samples to suggest that sleep duration is related to disinhibited eating behaviors, and short sleep may interact with these behaviors to promote excess weight [40,41]. However, although disinhibited eating is related to EAH, to date, there are no data among youth that examine the relationship between sleep and EAH specifically.

An important first step in understanding this potential relationship involves examining the association between overall average weekly sleep duration and facets of sleep (weekday, weekend, and weekend “catch-up” sleep) with EAH. Therefore, we examined the association of self-reported EAH with average weekly sleep duration, weekday (Sunday through Thursday nights) versus weekend sleep (Friday and Saturday nights), and weekend “catch-up” sleep (the difference between weekend and weekday sleep [42]) using an objective measure of sleep behavior among a sample of non-treatment seeking youth. We hypothesized that, after adjusting for relevant covariates (age, sex, race, height, total fat mass, and depressive symptoms), individuals with (1) less average weekly sleep, (2) fewer hours of weekday sleep, (3) more hours of weekend sleep, and (4) greater weekend “catch-up” sleep would report greater EAH. Further, we expected that, after adjusting for relevant covariates (age, sex, race, height, and depressive symptoms), individuals with (1) less average weekly sleep and (2) fewer hours of weekday sleep, (3) more hours of weekend sleep, (4) greater “catch-up” sleep, and (5) a higher reported EAH would have greater fat mass. Finally, in an exploratory analysis, we hypothesized that EAH would mediate the relationship between facets of sleep and fat mass such that fewer hours of average weekly or weekday sleep and greater “catch-up” sleep would lead to greater EAH and greater fat mass.

## 2. Materials and Methods

### 2.1. Participants

Healthy, non-treatment-seeking boys and girls (8–17 years old) were recruited to participate in the Children’s Growth and Behavior Study (Clinical Trials Identifier: NCT02390765), which was designed to understand eating behaviors in relation to body weight in children longitudinally. The protocol was approved by the Institutional Review Boards at the Eunice Kennedy Shriver National Institute of Child Health and Human Development and the Uniformed Services University. All participants were in good general health, with the exception of minor, well-controlled ailments. Exclusion criteria included (1) a history of major illness, brain injury, or pregnancy; (2) the use of medication known to affect body weight or energy intake; (3) significant and recent weight loss (>5% body weight); (4) BMI <5th percentile for age and sex; (5) current and regular use of illicit substances; (6) the presence of a full-threshold psychiatric disorder; and (7) a full scale intelligence quotient score <70 [43]. Signed informed consent and assent were obtained from parents/caregivers and children, respectively.

### 2.2. Procedure

Participants were seen at the outpatient Pediatric Clinic at the National Institutes of Health Hatfield Clinical Research Center. In order to evaluate overall health, a health history was taken, and children were examined by a physician or nurse practitioner. Following an overnight fast, height (cm) was measured in triplicate to the nearest millimeter by a calibrated stadiometer, and weight (kg) was measured using a calibrated scale after participants removed shoes and heavy clothing. BMI percentiles normed for age and sex were computed according to the Centers of Disease Control and Prevention growth standards, with overweight being defined as a BMI percentile 85th–<95th, and obesity defined as BMI percentile ≥95th [44]. Given that BMI is known to inconsistently capture differences in adiposity [45], we directly measured fat mass. Total lean and fat mass were measured using dual-energy X-ray absorptiometry (DEXA; iDEXA system, GE Healthcare, Madison, WI). As detailed below, participants completed questionnaires and were given wrist-mounted triaxial accelerometers to wear for a 14 day period on their non-dominant arm.

### 2.3. Measures

Sleep: Wrist-mounted triaxial accelerometers (ActiGraph LLC, Pensacola, FL, model GT3X) were configured based on height (cm), weight (kg), race, age, and handedness to collect information in 60 s epochs. Participants were instructed to wear accelerometers throughout 14 days and nights. They were asked to complete sleep diaries during this period, as well as to make notes of anything that might have significantly influenced their sleep (e.g., staying over at a friend’s house). To be included in analyses, participants were required to have valid data from ≥3 weekday nights of sleep and ≥1 weekend night of sleep. As described in Mi et al., 2019 [46], sleep data were downloaded from devices using Actilife software version 6.13.3 (Actigraph, Pensacola, FL). The Sedeh sleep detection algorithm, which has been previously validated for adolescents [47], was applied to the data in order to detect periods of sleep. Data were also visually inspected and confirmed by cross-referencing sleep diaries. In-bed time was defined as the first of a five-minute period in which activity counts were all zero and the subsequent activity counts were determined to be sleep by the Sedeh algorithm. Wake up time was similarly defined as the first of a five-minute period in which activity counts were above zero and the subsequent activity was determined by the Sedeh algorithm to be awake time. Average nightly sleep (min/night; total time in bed minus sleep onset minus minutes of wakeful activity after sleep onset) was also calculated. If a nap occurred less than 60 min from the wake-up time, the nap was included as part of the total nightly sleep. Naps (i.e., sleep that did not occur during part of total nightly sleep) were not included in analyses. Average weekly sleep duration (hr/night) of all nights (Sunday–Saturday) was calculated. Additionally, the average durations (hr/night) of weeknights (Sunday–Thursday), and weekend nights (Friday–Saturday) were calculated. Finally, weekend “catch-up sleep” was calculated by subtracting the average weekday sleep from average weekend sleep (hr/night) [42]. The term “catch-up sleep” is used in the literature for this weekend–weekday difference, even though there are participants who sleep less on the weekends [42]. Actigraphy has been previously validated for objective sleep duration measurement, with an overall agreement rate of >90% when compared to polysomnography (PSG) in normal adult subjects [48,49]. In a study done in healthy youth (5–12 years), actigraphy also demonstrated acceptable validity for average weekly sleep when compared to PSG [50].

Eating in the absence of hunger. EAH was measured with the 14-item Eating in the Absence of Hunger Questionnaire for Children and Adolescents (EAH-C [33]). Items assessed eating when not hungry or past satiation in response to external cues (e.g., “how often do you keep eating because others are still eating?”); fatigue and boredom (e.g., “how often do you keep eating because you are feeling bored”); and negative affect (e.g., “how often do you keep eating because you are feeling anxious or nervous?”). Items are rated on a 5-point Likert scale ranging from 0 = “never” to 4 = “always.” The average total score of all items on the EAH-C was used, with higher scores indicating greater EAH. The EAH-C has demonstrated convergent validity and temporal stability for all scales [33] and good internal consistency in the current sample (Cronbach’s α = 0.85).

Depressive symptoms. Depressive symptoms were assessed using the Children’s Depression Inventory, Second Edition [51,52,53]. This measure asks respondents to indicate to what degree they have experienced depressive symptoms in the last two weeks. Each item response ranges in order from 0–2, and total scores range between 0 and 52. Higher total scores indicate more depressive symptoms. The Children’s Depression Inventory- Second Edition is a widely used measure with good validity [54] and good internal consistency for the present sample (Cronbach’s α = 0.86).

### 2.4. Statistical Analyses

All analyses were conducted using SPSS version 25 for Windows (IBM Corp. Armonk, NY). Data were screened for outliers and normality. Neither EAH-C total scores nor depressive symptom total scores were normally distributed. Therefore, scores were log base 10 transformed to achieve normality.

Three multiple linear regressions were conducted to examine the associations of 1) average weekly sleep (hr/night), 2) average weekday and weekend sleep time, (entered into the same model; hr/night), and 3) weekend “catch-up” sleep (hr/night) with EAH-C total score. Four additional multiple linear regressions were conducted to examine the associations of 1) average weekly sleep (hr/night), 2) average weekday and weekend sleep time, (entered into the same model; hr/night), 3) weekend “catch-up” sleep (hr/night), and 4) EAH-C total score in relation to fat mass (kg). Because sleep timing is known to have associations with excess weight [55], eating related cognitive control [25], and reward sensitivity [29], in exploratory analyses, all tests were re-analyzed using weekday versus weekend in–bed time and wake times rather than sleep duration. For all models, given the links of depressive symptoms with sleep in children [56,57], the depressive symptoms score was entered as a covariate. Additionally, all models were adjusted for age, sex (0 = male, 1 = female), race (1 = Non-Hispanic white, 0 = other), height (cm). Finally, total fat mass (kg) was entered as a covariate in models that included EAH as a dependent variable. All tests were two-tailed and, *p* < 0.05 was considered significant. Using G*Power software version 3.1 [58], a power analysis was conducted to determine the sample size required for a small effect size (r^2^ = 0.14) with α = 0.05, power = 0.80, and the number of predictors as 7. The sample size needed was determined to be 96. Therefore, our sample was predicted to be adequately powered for such analyses.

Understanding that sleep differs across the age spectrum [14,15], secondary analyses were conducted to examine if patterns of results differed by age (child <12 years; adolescent ≥12 years). Although sample sizes were not adequately powered for statistical interpretation by age group, patterns of results did not differ from the total sample. Therefore, all subsequent results are represented with the age groups combined.

An exploratory mediation model was conducted using Preacher and Hayes Indirect Mediation macro for SPSS [59]. The model examined whether EAH mediated the relationship between facets of sleep and fat mass. Bootstrapping with 1000 resamples was used to estimate the 95% confidence interval for indirect effects.

## 3. Results

One-hundred-forty-eight children participated. Twelve youths were excluded from the analyses due to non-compliance with the actigraphy-wearing protocol, and ten were excluded due to technological issues downloading their actigraphy data. Those with missing actigraphy data did not significantly differ from the studied sample with regard to sex, race, age, or BMI*z* (*p*s > 0.22). One participant was excluded for missing depressive symptom data, and two participants were excluded due to missing DEXA data. Therefore, a total of 123 participants (52.0% female; 12.7 ± 2.6 years; 43.9% Non-Hispanic White; 30.9% with overweight/obesity) were included in the final analyses (Table 1). On average, from the 14 days of data collection, data were available for 13.3 (± 2.2) days of average weekly sleep, 9.5 (± 1.6) days of weekday sleep, and 3.8 (± 0.9) days of weekend sleep. Average weekly sleep was 7.3 ± 0.8 hr/night, average weekday sleep was 7.2 ± 0.8 hr/night, average weekend sleep was 7.5 ± 1.0 hr/night, and average weekend “catch-up” sleep was 0.3 ± 1.0 hr/night. Mean values were significantly different between samples with overweight and samples without overweight for race/ethnicity, fat mass, and BMI*z* scores, as expected.

### 3.1. EAH

**Figure 1:**
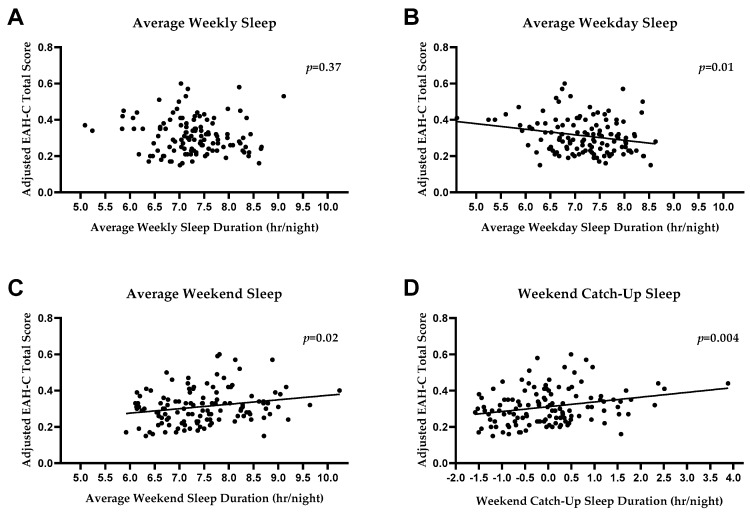
Associations between sleep and eating in the absence of hunger (EAH). There was no association between average weekly sleep and EAH total score (*p* = 0.37, **A**). There was a significant inverse association between EAH and average weekday sleep (*p* = 0.01, **B**), and a significant positive association between average weekend sleep and EAH (*p* = 0.02, **C**). There was a significant positive association between weekend “catch-up” sleep and EAH (*p* = 0.004, **D**). All analyses were adjusted for age, sex, race, height, total fat mass, and depressive symptoms.

Adjusting for covariates, average weekly sleep duration (hr/night for the entire week) was not significantly associated with EAH (F (7115) = 1.57, β = −0.09, *p* = 0.37, R^2^ = 0.09, Figure 1A). However, weekday average sleep time (hr/night) was inversely associated with EAH (F (8114) = 2.46, β = −0.25, *p* = 0.01, R^2^ = 0.15; Figure 1B) such that participants with greater weekday sleep reported lower EAH. Conversely, weekend average sleep time (hr/night) was positively associated with EAH (F (8114) = 2.46, β = 0.23, *p* = 0.02, R^2^ = 0.15; Figure 1C), such that participants with more weekend sleep reported greater EAH. Finally, weekend “catch-up” sleep (hr/night) was positively associated with EAH-C total score (F (7115) = 2.79, β = 0.26, *p* = 0.004, R^2^ = 0.15, Figure 1D), such that individuals with greater weekend “catch-up” sleep reported greater EAH. In exploratory analyses, neither weekday (F (8114) = 1.61, β = −0.09, *p* = 0.38, R^2^ = 0.10) nor weekend (F (8114) = 1.61, β = 0.18, *p* = 0.11, R^2^ = 0.10) in-bed time was significantly associated with EAH. Similarly, neither weekday (F (8114) = 1.36, β = −0.06, *p* = 0.61, R^2^ = 0.30) nor weekend (F (8114) = 1.36, β = −0.04, *p* = 0.71, R^2^ = 0.30) wake time was associated with EAH.

### 3.2. Fat Mass

Adjusting for age, sex, height, race, and depressive symptoms, none of the facets of sleep (average weekly F (6116) = 3.76, β = −0.07, *p* = 0.42, R^2^ = 0.16; weekday F (7115) = 3.76, β = 0.001 *p* = 0.99, R^2^ = 0.17; weekend F (7115) = 3.79, β = −0.12, *p* = 0.22, R^2^ = 0.18, or weekend “catch-up” F (6116) = 3.79, β = −0.08, *p* = 0.38, R^2^ = 0.16) were significantly associated with fat mass. Moreover, EAH-C total score was not significantly associated with fat mass (F (6116) = 3.74, β = −0.06, *p* = 0.47, R^2^ = 0.16). In exploratory analyses, neither weekday (F (7115) = 3.55, β = −0.17, *p* = 0.12, R^2^ = 0.18) nor weekend in-bed time (F (7115) = 3.55, β = 0.0.05, *p* = 0.68, R^2^ = 0.18) was significantly associated with fat mass. Similarly, neither weekday (F (7115) = 3.12, β = 0.05, *p* = 0.66, R^2^ = 0.16) nor weekend (F (7115) = 3.12, β = −0.01, *p* = 0.91, R^2^ = 0.16) wake time was associated with fat mass.

### 3.3. Exploratory Mediation Analyses

EAH did not mediate the relationship between any facet of sleep [average weekly (R^2^ = 0.01; 95% bootstrap CI: (−0.004 to 0.009); SE = 0.003), weekday (R^2^ = 0.01; 95% bootstrap CI: (−0.005 to 0.010); SE = 0.004), weekend (R^2^ = 0.007; 95% bootstrap CI: (−0.005 to 0.004); SE = 0.002), weekend “catch-up” (R^2^ = 0.004; 95% bootstrap CI: (−0.009 to 0.007); SE = 0.004] and fat mass (Table 2).

## 4. Discussion

Using actigraphy, an objective measure of sleep duration, we found no significant association between average weekly sleep duration and self-reported EAH. However, when sleep duration was examined separately for weekdays and weekends, significant associations emerged, such that weekday sleep was negatively associated with EAH, weekend sleep was positively associated with EAH, and, correspondingly, weekend “catch-up” sleep was positively associated with EAH.

The finding that there was no significant association between average weekly sleep and EAH was contrary to our hypothesis. It is possible that examining sleep as a continuous variable rather than categorically by sufficiency attenuated the strength of this association. Indeed, previous literature has often categorized youth sleep duration into “inadequate” or “adequate” when examining it in relation to weight outcomes [6]. Studies suggest that sleeping too little (less than eight hours per night) or too much (twelve or more hours per night) may be more robustly associated with eating behaviors and obesity risk than average sleep duration alone [60,61,62,63]. However, given the lack of specified hours of nightly sleep recommended for certain age ranges [6], and the lack of individuals in the present sample with 12 h or more of sleep, the present study analyzed the sleep data continuously rather than categorized by sleep sufficiency and adjusted for age.

Notably, both weekday and weekend sleep were significantly associated with EAH. EAH was inversely associated with weekday sleep, but positively associated with weekend sleep and weekend “catch-up” sleep. It appears that examining group means obscures the individual differences in weekday and weekend sleep, which are reflected in the wide range of the Weekend Catch-Up Sleep Duration in Table 1 (−1.5 to +4.5 h). These findings highlight the potential importance of sleep variability. A study by He et al. [38] made use of objective measures of sleep and found that habitual sleep variability (intra-subject standard deviation of average sleep duration across all 7 nights of sleep) was significantly associated with abdominal adiposity among adolescents. Notably, however, He et al. found no significant association between total average sleep duration and abdominal adiposity. Such findings are consistent with other reports, indicating that sleep variability may be associated with eating behaviors and excess weight over and above sleep duration alone [25,26,27,38,39]. Our results complement and build on this literature, in that even small differences in sleep duration between weekday and weekend may have unique associations with disinhibited eating behaviors.

Similarly, our findings indicate that children with greater weekend “catch-up” sleep reported greater EAH. This supports previous literature, which suggests that weekend “catch-up” sleep may be uniquely associated with reward-based disinhibited behaviors due to circadian rhythm misalignment [28]. Although there are few studies to date examining the link between circadian rhythm shift and eating behaviors among youth, a recent experimental study found that adults who worked night shifts are more likely to choose a high-fat breakfast option than those who worked regular daytime hours [64]. Interestingly, Depner et al. [65] recently reported that ad libitum weekend recovery sleep could not prevent metabolic dysregulation caused by experimentally induced insufficient sleep in young adults. These findings highlight the potential impact of circadian rhythm shift not only on disinhibition, but also on dietary intake and the metabolic consequences of short sleep. Data further suggest that sleep restriction and circadian misalignment are associated with decreased concentrations of leptin, an appetite hormone important for satiety, and with increased concentrations of ghrelin, an appetite-stimulating hormone [66,67,68,69,70]. It is possible that youth experiencing a shift in their biological circadian rhythm by engaging in weekend “catch-up” sleep [71] may create hormonal changes associated with increases in consummatory behaviors. Future research is warranted to assess the impact of weekday versus weekend sleep, weekend “catch-up” sleep, and circadian misalignment on these hormones.

We did not find a significant association for any sleep variable or EAH with fat mass. The extant literature is mixed regarding the association of sleep and EAH with adiposity [21,26,31,38,66,72,73]. Although neither sleep nor EAH was associated with fat mass, the significant association of weekend and weekday sleep with EAH may highlight an important risk factor for the development of excess weight gain over time. In exploratory analyses, EAH also did not significantly mediate the relationship between any of the facets of sleep examined and fat mass. This may be due to the relatively small sample size, as previous studies that have observed a relationship between objectively measured sleep and weight have had larger samples [21,26]. Further, data support that other dimensions of sleep, such as poor sleep quality and evening chronotype (preference for later bedtime) are associated with an increased risk of excess weight in youth [21,73,74,75]. It is possible that these factors, which were not measured in the present study, are more robustly associated with fat mass than nightly hours of sleep. Longitudinal data from large samples are needed to investigate these relationships further.

Study strengths include the use of actigraphy to analyze participants’ sleep, which provided an objective measure of a difficult variable to quantify by self-report. Additionally, our study included a racially and ethnically diverse sample of healthy youth with a broad range of ages and weights. Finally, we utilized a widely used self-report measure of EAH [33].

Limitations of this study include the use of cross-sectional data and, thus, no causal or mechanistic conclusions can be drawn from findings. An additional limitation is that our sample was not large enough to examine the variability of sleep needs across development [15], and the range of sleep was limited. Similarly, our sample did not have enough participants with overweight or obesity to analyze results by weight status. Given the known associations between sleep duration and EAH with weight status [7,19,21,22,23,31,32], future studies should recruit individuals specifically with overweight and obesity in order to better elucidate any potential differences among groups. Additionally, given the nature of the EAH-C as a self-report measure, it may not reflect actual energy intake [76,77]. However, the measure is positively associated with daily energy intake among obesity-prone youth [76] and, therefore, may highlight a disinhibited eating behavior that places youth at risk for excess weight. Future studies should investigate sleep variability in relation to EAH assessed in the laboratory. Additional research should also assess these associations among samples that specifically recruited individuals with overweight and obesity. Finally, future studies should assess chronotype in order to effectively examine the associations of chronotype and circadian misalignment with EAH and fat mass.

In conclusion, this study builds on previous literature to suggest that sleep characteristics such as weekday versus weekend sleep and weekend “catch-up” sleep may be associated with higher self-reported disinhibited eating behavior [25,26,27]. Prospective data are needed to determine if this association contributes to the relationship between sleep and excess weight gain risk, as EAH and sleep variability may be modifiable targets for reducing and preventing the high rates of pediatric obesity.

## Figures and Tables

**Table 1:** Sample characteristics.

Characteristic ^a^	Sample with Overweight (*n* = 38)	Sample without Overweight (*n* = 85)	Total Sample (*n* = 123)	Total Sample Range
Age (years) Sex (% female) Race/Ethnicity (%) * Hispanic or Latino Asian Black or African American Multiple Races White Unknown Fat Mass (kg) * Overweight/Obesity (≥ 85th percentile, %) BMI*z* Score * Average Weekly Sleep Duration (hr/night) Weekday Sleep Duration (hr/night) Weekend Sleep Duration (hr/night) Weekend “Catch-Up” Sleep Duration (hr/night) Eating in the Absence of Hunger	12.4 ± 2.5 58.0 7.9 0.0 47.4 10.5 31.6 2.6 24.9 ± 9.4 1.7 ± 0.4 7.1 ± 0.8 7.0 ± 0.8 7.3 ± 1.0 0.2 ± 0.9 1.1 ± 0.5	12.9 ± 2.6 49.0 7.1 16.5 21.1 5.9 49.4 0.0 11.2 ± 4.4 0.0 ± 0.7 7.3 ± 0.7 7.2 ± 0.8 7.6 ± 1.0 0.3 ± 1.0 1.1 ± 0.5	12.7 ± 2.6 52.0 7.3 11.4 29.3 7.3 43.9 0.8 15.5 ± 9.0 30.9 0.5 ± 1.0 7.3 ± 0.7 7.2 ± 0.8 7.5 ± 1.0 0.3 ± 1.0 1.1 ± 0.5	8.0–17.9 4.0–54 −1.6–2.8 −1.6–2.8 4.9–8.8 4.6–8.7 5.3–9.6 −1.5–4.5 0.5–3.0

^a^ Values presented are mean ± standard deviation, unless otherwise noted as percentage. Results are presented for individuals with/without overweight to examine if there are any significant differences in outcome variables between the two groups. * Mean values were significantly different between the sample with overweight and the sample without overweight for race/ethnicity, fat mass, and body mass (BMI)*z* scores, as expected; *p* < 0.05.

**Table 2:** Results of Mediation Model.

Facet of Sleep	Direct Effect (*c’* Path)			Indirect Effect (*ab* Path)		
	*t*-Value	SE	*p*-Value	95% CI	R^2^	SE
Average Weekly Sleep Weekday Sleep Weekend Sleep Weekend Catch-up Sleep	−1.33 −1.30 −0.90 0.02	0.02 0.02 0.01 0.01	0.19 0.20 0.37 0.85	−0.004–0.009 −0.005–0.01 −0.005–0.004 −0.009–0.007	0.01 0.01 0.007 0.004	0.003 0.004 0.002 0.004
